# Access to Health Services and Assistance Offered to the Afro-Descendant Communities in Northern Brazil: A Qualitative Study

**DOI:** 10.3390/ijerph18020368

**Published:** 2021-01-06

**Authors:** Marcela de Oliveira Feitosa, Maria Elidiana Araújo Gomes, Iolanda Graepp Fontoura, Catilena Silva Pereira, Ana Maria da Costa Teixeira Carneiro, Maikon Chaves de Oliveira, Janayna Araújo Viana, Volmar Morais Fontoura, Késia Chaves da Silva, Renata de Sá Ribeiro, Paula Cristina de Sousa Vieira, Sarah Gisele de Vasconcelos Leite, Lígia Parente de Alencar Leal, Ankilma do Nascimento Andrade Feitosa, Fernando Luiz Affonso Fonseca

**Affiliations:** 1 Department of Postgraduate, ABC Faculty of Medicine, ABC University Center, Santo André, 09060-650 São Paulo, Brazil; maria.elidiana@hotmail.com (M.E.A.G.); profferfonseca@gmail.com (F.L.A.F.); 2 Department of Nursing, Federal University of Maranhão, Imperatriz, 65915-240 Maranhão, Brazil; iolandagraepp@hotmail.com; 3 Department of Nursing, State University of Tocantins, Augustinópolis, 77960-000 Tocantins, Brazil; catilena.sp@unitins.br (C.S.P.); ana.leka@hotmail.com (A.M.d.C.T.C.); maikonchaves@hotmail.com (M.C.d.O.); janayna.av@unitins.br (J.A.V.); volmar_morais@hotmail.com (V.M.F.); kesia.chaves@hotmail.com (K.C.d.S.); renatadesaenf@outlook.com (R.d.S.R.); 4 Higher Education Unit of South Maranhão, UNISULMA, Imperatriz, 65907-070 Maranhão, Brazil; 5 Federal Institute of Roraima, West Zone Boa Vista, 69318-070 Roraima, Brazil; paula.vieira@ifrr.edu.br; 6 Surgery and Experimental Research Program, State University of Pará (UEPA), 66095-661 Belém, Brazil; sarahgisely@hotmail.com; 7 Hospital Regional do Sertão Central—HRSC, Quixeramobim, 63800-000 Ceará, Brazil; xliginhalealx@hotmail.com; 8 Nursing and Medicine Department, University Santa Maria, Cajazeiras, 58900-000 Paraiba, Brazil; ankilmar@hotmail.com; 9 Department of Pharmaceutical Sciences, Federal University of São Paulo (SP), 09913-030 Diadema, Brazil

**Keywords:** health care, public health policies, vulnerable communities, group with ancestors from the African continent

## Abstract

The remnants of quilombos, individuals of African descent, have faced several barriers throughout its history, either due to prejudice imposed by society, or the non-fulfillment of their rights guaranteed in the 1988 Constitution, such as access to health services. Thus, this study aims to evaluate the health care offered to quilombo communities in the northern region of Tocantins. This is an exploratory, descriptive study with a qualitative approach, including field research and focus group, carried out with 58 quilombo remnants people from communities in the northern region of Tocantins. Data collection was carried out between from October 2017 to July 2018, through semi-structured interviews. We found that these communities have limited access to health services, in addition to a negative perception of the assistance offered to the health of their population and the commitment of managers. Therefore, access to health and assistance received by the studied communities required to be prioritized since the care provided is not unique and has not met the health demands and needs of the remaining quilombos in northern Tocantins, Brazil.

## 1. Introduction

In Brazil, the period of slavery was between the 16th and 19th centuries. At that time, black individuals from different regions of the African continent were brought by force from their country of origin in ship holds to be marketed as slaves and to carry out heavy work in agriculture, in mines, sugar mills, and with excessive hours [1,2]. The transatlantic traffic allowed the slavery of blacks in Europe, Asia, the Americas and even Africa [3], where 11 million were forcibly taken to the American continents (North and South) and 4.9 of these remained in Brazil. Brazil was the country that retained the largest number of slaves [4].

The enslaved black people were socially marginalized, treated inhumanly, persecuted, punished, and tortured by those who bought them [1,2]. They also did not have access to fundamental rights, including health, education, decent housing, and leisure [4]. For this reason, well-being, health, and quality of life were compromised and the life expectancy of this population was reduced [5,6].

In this scenario, the slaves began to escape, and inhabit areas with difficult access called quilombos. Quilombos became the greatest strategy of opposition to the slave system, eradicated on 13 May 1888, with the publication of the Golden Law by Princess Isabel [2]. After conquering freedom, their fundamental rights were ensured by the Federal Constitution (FC) of 1988, and also recognized as an ethnic–racial group. The communities they formed were called Quilombos Remaining Communities (QRCs) through article 68 of the FC [7].

Currently, QRCs are composed of Afro-descendant individuals who declared to be remnants of quilombos, remaining in the areas inhabited by their ancestors and maintaining the customs and traditions of the African continent, which are also called quilombolas [8]. The Palmares Cultural Foundation (*FCP*) point out that, up to December 2015, 2620 quilombo communities were certified in Brazil, and 44 of them were located in Tocantins [9,10], the newest state that is located in the northern Brazilian region. In 2018, *FCP* recognized 338 more communities, totaling 2958 remaining quilombo communities in the country [11].

Brazil occupies the fifth largest population in the world, estimated at 211,293 million inhabitants, classified as white (45.22%), brown (45.06%), black (8.86%), yellow (0.47%) and indigenous (0.38%). The Brazilian black population is constituted by blacks and mediums brown (53.92%). This combination is statistically justified by the fact that these two groups present similar socioeconomic aspects and, theoretically, because they are targets of discrimination.

[12,13,14] (p. 6, 2020).

The report published by the World Bank in 2018, states that Brazil occupies the second position in the ranking of countries with the highest number of people of African descent, with about 105 million Afro individuals, including quilombolas, and Nigeria is the first in the ranking of those countries with greater number of afro-descendants [3]. In terms of public policies for this ethnic group, the National Policy for the Promotion of Racial Equality (*PNPIR*) was created on 20 November 2003, through Decree No. 4.886/2003 and the National Policy for the Integral Health of the Black Population (*PNSIPN*) [15]. *PNPIR* aims to reduce race asymmetries that exist in the country and that affect, especially, the black population, including quilombos communities [16]. The Ministry of Health also reinforced equal, universal, efficient, and comprehensive care for these populations when it published Ordinance No. 992/09, on 13 May 2009, which establishes the *PNSIPN* [17].

In this context, the Brazilian Unified Health System (SUS), created in 1988, also stands out, being, therefore, a great social achievement, which recognized health as a fundamental right and duty of the state to guarantee integral and universal access to health, taking into consideration social and economic conditions of a certain population and not only the ethnicity. The implementation of SUS has contributed to health care to the population in the most different aspects [18].

However, even though these policies exist, quilombolas continue to be victims of prejudice, xenophobia, racial discrimination, and social inequities, and access to health services [19,20,21]. Studies report that these inequalities are linked to ethnic and race issues experienced more frequently by black, brown, and indigenous people [22,23,24]. A recent example is the pandemic of the new Coronavirus, called SARs-COV19, which affected this population and demonstrated even more the inequalities in access to health, since many remaining quilombolas were infected due to lack of access to information and because they live in isolated areas with precarious sanitary conditions and infrastructure in their communities, especially the limited access to health services [25].

Research in the United States emphasizes that African-American individuals have less access to health services than individuals of other races [26,27]. For this reason, the Organization of American States (OAS) together with the Pan American Health Organization (PAHO), proposed in 2016, a health plan for the more than 150 million Africans that inhabit the different countries of the Americas. The aforementioned OAS and PAHO plan established from 2015 to 2024 aimed at ensuring greater access to health services for people of African descent and, consequently, more health, well-being, and quality of life [28].

In Brazil, Afro-descendants, including the remaining quilombos, have less access to health services at different levels of health care, mainly in primary health care, the gateway to the Unified Health System, restructured through the Family Health Strategy (FHS), responsible for developing health promotion, prevention, and restoration actions [24,29]. The performance of FHS Teams (FHt) in quilombola communities was emphasized in the National Policy for Primary Care (*PNAB*), published by Ordinance No. 2436, on 26 September 2017 [30], to expand this group’s access to health services and guide them in facing the SDH and guarantee access to health with a fundamental right [27].

Starting from the premise that health and a right for all, several concerns about the assistance offered to the remaining quilombo communities in Brazil emerge. Thus, the scarcity of studies in northern Brazil, especially in the state of Tocantins, highlights the difficulties faced by quilombolas to access health services, and the assistance they receive when they need health care, making it a pioneering study.

Therefore, this study aims to assess access to health services and assistance offered to remain quilombo communities in the northern region of Tocantins, Brazil.

## 2. Materials and Methods

### 2.1. Design

This is an exploratory, descriptive study, with a qualitative approach, cross-sectional and including field research in remaining quilombo communities in the northern region of the State of Tocantins, through a focus group and semi-structured interview. The study aimed to observe, explore, and describe the proposed theme.

The focus group is an interview technique applied in groups, based on communication and interaction to raise detailed data about a theme proposed by the researcher, after selecting the participants to compose the group and start the questions. The focus group aims to acquire data that enable the researcher to understand perceptions, beliefs, attitudes about a certain topic, object, or services [31].

The focus group is a technique of approach that promotes interaction between the group, and each participant answers the questions made by the interviewer, in order to expose their point of view, their knowledge, their desires on the topic addressed. In addition, it is highlighted that the dynamics and involvement of the group will help the researcher in the analysis of the data obtained on the topic discussed [32].

### 2.2. Study Participants and Inclusion and Exclusion Criteria

The state of Tocantins is located in the North and its capital is Palmas, having 139 municipalities divided into 8 microregions, which are Bico do Papagaio (25 municipalities), Araguaína (17 municipalities), Miracema do Tocantins (24 municipalities), Jalapão (15 municipalities), Porto Nacional (11 municipalities), Rio Formoso (13 municipalities), Gurupi (14 municipalities), and Dianópolis (20 municipalities). The state of Tocantins borders the state of Maranhão in the northeast, Piauí in the east, Goiás in the south, Bahia in the southeast and Mato Grosso in the southwest, and Pará in the north. The state covers an area of 277,620 km^2^ [33].

The state of Tocantins has 44 communities certified as quilombos remnants [10]. However, due to the scarcity of studies on the living conditions and health of these communities, mainly those located in the Northern region of Tocantins, due to the following limitations: geographical location unfavorable since most communities belong to the rural zone and are isolated from the urban center; poor road conditions; lack of transport; resistance from the communities, both for the persistent struggle for their rights, and to be recognized as an ethnic–racial group, in addition to the prejudice imposed by society throughout the history of its predecessors. Such conditions constitute barriers to the acceptance, authorization and participation of the remaining quilombos of Brazil in different studies.

Therefore, the present investigation was carried out in six communities in the northern region of Tocantins, Brazil: São Vicente Island located in the municipality of Araguatins—TO; Carrapiché, Prachata, and Ciriáco, belonging to the municipality of Esperantina; Grotão located in the rural area of Filadélfia—TO; Cocalinho and surroundings belonging to the municipality of Santa Fé do Araguaia in six communities that are located in the northern region of that State: Ilha de São Vicente, Carrapiché, Prachata, Ciriáco, Grotão, Cocalinho, and surroundings.

The quilombola community Ilha de São Vicente is located in the municipality of Araguatins-TO. The access to the community is by the Araguaia river, and it is only possible to go by boat taking about 20 min or more, as it depends on the type of engine and the vessel used [34].

The quilombola communities Prachata, Ciriáco, and Carrapiché belong to the municipality of Esperantina, certified by the Fundação Cultural de Palmares in 2015, although they have been recognized as remnants of quilombos for many years. The Ciriáco quilombola community is eight kilometers and Carrapiché is 14 km from the urban area of Esperantina. The quilombola community Prachata is located on an island in the middle of the Tocantins River, known as Ilha do Mel, far from the urban area going in the river by boat.

The quilombo community Grotão is located in the rural area of the municipality of Filadélfia, approximately 82 km from the urban area of that municipality. The community of Cocalinho and surroundings was recognized by the Fundação Cultural de Palmares in 2006 [35]. The Cocalinho community and its surroundings are located about five kilometers from the municipality of Santa Fé do Araguaia.

The inclusion criteria adopted in this study were the remaining quilombolas with a minimum age of 18 years old and a maximum of 70 years old, of both genders, and living in the quilombola communities where the study was conducted. The exclusion criteria were quilombolas with psychocognitive restrictions, which could interfere in the understanding of the theme studied.

Sampling was determined using the convenience technique, considering individuals in the community who were available at the time of data collection and who agreed to participate in the study. The sample size was defined using the saturation technique, for reaching the saturation of the data with the study sample and for not interfering in the understanding of the phenomenon by the researcher. The technique of saturation does not require the performance of calculation or the use of a certain mathematical formula, to justify in a probabilistic way its application [36].

Moreover, in this technique, the number of participants is operationally defined as the suspension of inclusion of new participants, when the data obtained start to present, in the evaluation of the researcher, certain redundancy or repetition not being considered productive persist in the collection of data

[37] (p. 41).

Thus, 58 remnants of quilombola communities from the northern Tocantins participated in the study, with 12 remnants of the quilombola community of Ilha de São Vicente; 10 from the Ciriáco community; 9 from the Prachata community; 9 from the Carrapiché community; 12 from the Cocalinho community, and 6 from the Grotão community.

### 2.3. Data Collection

Data collection was carried out between October 2017 and July 2018, after approval by the Ethics and Research Committee of the ABC Faculty of Medicine. Data were obtained through semi-structured interviews, with 6 focus groups, one in each community surveyed, with a duration of 120 min and an average of 9 participants per focus group, most of them female, because they are always in the community, because their spouses need to leave the territory to go for farming or fishing and, for this reason, many return only at the end of the day, or at the end of the week. Fishing and agriculture are the main sources of income for the population studied.

The interview script contemplated personal and inherent information about the perception of the study participants about the concept of health, as well as, about the main health problems faced by their community, the health care received and the commitment of managers in terms of health, having as guiding questions: What is health? Does the community receive assistance in the territory? Are they satisfied with the health care offered to the population of their community? How do you evaluate the managers’ commitment?

It is worth mentioning that 58 individual interviews were carried out, being this sample composed by 42 women and 16 men. Individualized interviews were conducted during home visits, on days previously scheduled with the leaders of the communities, and lasting 30 min for each participant, for being the average time to apply an interview and for following a previously established script, which allows the interviewee to expose his or her perceptions on the subject approached, with minimal interference from the researcher (Table 1).

The interview script included personal information and inherent to the study participants’ perception of the concept of health, and about the main health problems faced by their community, the health care received, and the managers’ commitment in terms of health. In focus group interviews, the moderator should present a good script to conduct the group discussion, which not only allows a progressive deepening (funnel technique), but also the fluidity of the debate without the need to intervene several times [31].

The focus group meetings were held in the association’s shed in the Ilha de São Vicente community, Ciriáco, and in the other communities, they were held in the leadership house by not having an appropriate space. The dialogue in the group started after all participants had read and signed the Informed Consent Term, agreeing with their participation in the research and knowing their objectives.

Their speeches were identified by one or two letters followed by a number, such as P1 to respect the ethical aspets of research involving human beings and ensure the anonymity of the study participants. The letters P, Ci, Ca, Co, G, and I are the initials of the quilombola communities studied, Prachata, Ciriáco, Carrapiché, Cocalinho, Grotão, and Ilha de São Vicente and the number represents the order in which the interviews were conducted. The interviews were recorded and transcribed in full to be compared with each other and discussed, based on the literature.

### 2.4. Data Analysis

The collected data were analyzed using Laurence Bardin’s Content Analysis technique, which seeks to describe the perceptions and reactions contained in the statements [38] of representatives of quilombola communities in the state of Tocantins, regarding health care offered to their communities and managers’ commitment to this population, respecting the individuality of each one.

### 2.5. Ethical Aspects

The procedures adopted in this research obeyed the Criteria of Ethics in Research with Human Beings according to Resolution Nº. 466/2012 of the National Health Council. This resolution was read exhaustively by the researchers to follow it and respect it in its entirety and complexity.

This study was approved by the Ethics and Research Committee of the ABC Medical School under Opinion N°. CAAE 74041317.8.0000.0082.

## 3. Results

The main objective of this study is to evaluate access to health services and assistance offered to remain quilombo communities in the northern region of Tocantins, Brazil. Thus, we organized the results into five categories and their subcategories, described in Table 2:

### 3.1. Characterization of Study Participants

From the results, we observed that most of the study participants, 42 (72.41%) are female, with a predominant age range between 41 and 50 years old, 19 (32.76%), with no participants aged 18 to 20 years old. In the marital status, 28 (48.27%) most of those surveyed are married.

Twenty-one of the 58 respondents have incomplete Elementary School, 06 are not literate, 12 have Complete Elementary School, totaling 39, which demonstrates the predominance of low education among the population studied, as only 6 participants have complete higher education, 11 complete high schools, and 2 incomplete high schools.

Regarding the occupational situation, most participants are fishermen (37.93%), with a minimum wage income (48.28%), and live with two to three people in the same house (43.10%). We also identified that most of them receive benefits from the government, such as family allowance, fisherman’s salary, and retirement.

As for the infrastructure of the communities, it was evidenced that most of them do not have sewage network, nor septic tank and sink, piped water, except for the community of Cocalinho and surroundings that the water comes from the well that exists in the community. In addition, it was noted that the water used by most communities from the Araguaia (São Vicente and Ciriáco) and Tocantins (Carrapiché and Prachata) rivers is not treated at all. Additionally, it is noteworthy that garbage is burned in most communities.

### 3.2. Knowledge about the Concept of Health

When seeking to know the concept of health in the perception of the study participants, we found that they presented a positive concept of health, as they expressed opinions, which are close to the concept published by the World Health Organization, that health “is a state of complete physical, mental and social well-being and not just the absence of a disease” [39], as shown in the following statements:

“Health is the person living well, eating well. In my understanding as a quilombo, owner of my land, health is eating without any product that has contamination, which is the products that are killing us, these old foods, full of poison, our people would die at 100 years old and I didn’t have these diseases: leprosy, cancer, these things”.

(G1)

“Health is to have a healthy diet, to have medical care without bureaucracy, also to have drinking water because, in the community, we drink water from the river without treatment and in the city, it is also not good because it is a little dirty. And those who have a car or motorcycle will fetch it from a well in Pedra Branca, which is eight kilometers from the city”.

(Ca3)

“For me, health is having the right to good health, the health worker comes to visit, to receive information. There are a lot of malaria cases and nobody from the health department came to give information”.

(Ci9)

We also found that some study participants perceive and conceptualize health as an essential condition for life:

“Health is everything, but a lot is missing, with the administration that we are having recently, it is leaving a lot to be desired, as equipment and other things are lacking”.

(Co3)

“Health is very important, for me, it comes first because without health, we are nothing”.

(Co10)

“Health means having a stable life, walking and seeing, I think that’s it. If you don’t, you don’t live”.

(I4)

“Health is everything, it is the hope that we have to live”.

(P7)

### 3.3. Most Frequent Health Problems

When asked about the most frequent health problems among people in the community, the speeches showed that Systemic Arterial Hypertension (SAH) was the pathology most reported by the population studied. We also noticed that Diabetes Mellitus (DM) was the second most pronounced pathology in the interviews. The statements below reveal these findings:

“All patriarchs, elders have diabetes, my parents also have diabetes and high blood pressure. My mother-in-law, my uncle, also has it. My uncle even had a stroke because he didn’t take any medicine and his pressure increased and he is dragging a leg”.

(G1)

“Heart problem, high blood pressure, diabetes”.

(I4)

“High blood pressure, cholesterol”.

(Ca1)

“Diabetes, high blood pressure, and cholesterol”.

(P2)

“High pressure”.

(Ci10)

In this category, in addition to SAH and DM, sickle cell anemia was another pathology mentioned by one of the remnants of the Cocalinho community:

“The biggest health problem in the community is high blood pressure, as we have 54 people with high blood pressure in the community, 15 with diabetes, and seven people who have both diseases, and six people with sickle cell anemia”.

(Co3)

### 3.4. Access to Health Services and Health Care

Regarding access to health services and health care, the following subcategories were identified when analyzing the statements of the study participants: lack of assistance in the territory, difficulties in accessing health services, fragmented assistance, lack of commitment from managers, professionals do not adequately perform their duties.

We also found that only the community of Cocalinho and its surroundings has a Family Health Team (FHt), which assists twice a week in the territory and the others do not receive any assistance within the community, as shown by the statements:

“When we get sick, we have to go to the health center in Araguatins because on the island, no one comes to see us”.

(I1)

“There is no health team here, we do not receive assistance within the community, if there were, I think things here were healthier and, when one of us gets sick, it is very difficult to get to the city”.

(G2).

“We have no assistance here”.

(Ci10)

“When there were elderly people in the community, they came to take the pressure and visit, but God took everything and they don’t even show up here”.

(P3)

“We have care in the community because the doctor is here every Monday, the dentist also comes and the nurse comes once or twice a week”.

(Co11)

However, remnants of the community of Cocalinho and surroundings highlighted that the service once or twice a week was not enough to meet the health demands of the population and, for this reason, they needed to travel to the municipality of Santa Fé from Araguaia in search of assistance, as evidenced by the statements:

“The doctor only comes once and the number of hypertensive, diabetic, and elderly people in the community is not enough to assist us”.

(Co2)

We also identified that the assistance offered to quilombolas in the North of Tocantins is fragmented, as it is restricted to medical consultation in the FHS, prescription of medication, consultation with the nurse and requesting tests, characterizing the curative model, centered on the disease and not in the biopsychosocial dimension of the individual, as shown in the following reports:

“My sister here, we go to the health center and if they don’t manage to take it to Augustinópolis. So, here in Araguatins, you can see that at no time, health is assisting with anything, even for those who live in the city”.

(I4)

“We go to the health center in Esperantina, we get there, then the doctor is called and we are treated; when it is serious, they take us to Augustinópolis, which is 80 km from the city”.

(Ci10)

“We go to the health center and the doctor gives the medicine”.

(Ca7)

“We did not receive a visit from the doctor and a nurse even to talk”.

(Ca2)

We also found that only the remnants of the community of Cocalinho and surroundings referred to participation in health promotion actions and that they received a home visit from the doctor and/or nurse. This finding was summarized in the following statement:

“In the basic health unit, they measure people’s pressure, weigh and then we consult with the doctor or nurse; sometimes, they go to the daycare center to assist the children and they weigh, measure, give vaccines, give them worm medicine, teach them how to brush their teeth, talk about food, hygiene”.

(Co1)

The negative perception of the study participants about health care was notorious since most of them assessed it as terrible.

“It terrible, because we don’t receive assistance in the community and the city there is only one health center and it doesn’t meet the demand”.

(P4)

“It’s sad. We have no responsible health team to do this work in the community and now we are without a health agent as well”.

(I4)

In the health care category, we also identified that pregnant women, the elderly people, hypertensive patients, diabetics, and children in the studied communities do not receive adequate, humanized, and resolving assistance, as shown in the following statements:

“Prenatal care is done at the health center that is 37 km from the community, which is the nearest town of Bielândia and these pregnant women have to go there by motorbike to consult themselves because no one comes to visit them in the community. When they ask for exams, they have to go to Philadelphia to make an appointment that it is 70 km from the community and it is a struggle to get it”.

(G1)

“Pregnant women in the community have to go to the Cesp, which is located in the city, and there go to the nurse, doctor if they need a vaccine, and there is also a nutritionist”.

(I7)

“The children of the community still do not have that specific care that is needed, when someone gets sick with a stomach ache, they will have to go there at the health center that is 8 km away”.

(Ci10)

“No, it is difficult here because it is far from the street and we cannot take it there, and nobody comes here, because they do not care for us and that is wrong”.

(Ca7)

“The elderly do not receive care at home, only if it is at the health center to make an appointment to see the doctor”.

(Ca4)

Furthermore, the study participants exposed several difficulties configured as a barrier to access to health services. Therefore, in this subcategory, the following registration units were identified: inadequate number of professionals, shortage of medicines and basic materials, lack of transportation, poor road conditions, obstacles for making the appointment, carrying out exams, and receiving results, such as shown in the statements:

“Here, there is a lack of doctors, medicine, and roads that are not good for going to the city to consult”.

(Ci10)

“There is no direct doctor and we need to go at dawn to get an assistance card”

(P2).

“Lack of boats and transportation to take us to the city”.

(I2)

“We have difficulties to do the exams because the professional who attends us asks and we book, but some only do it in Araguaína and it takes from one to three months and, if we don’t have the money to go, we don’t do it”.

(Co10)

“When we go to the health center, sometimes we pay, even to have a diabetes test”.

(G1)

Therefore, the distance from the communities is one of the main barriers of access to the studied population to health services since the quilombolas of the Grotão community need to cover 37 km to receive assistance. Many times, they cannot get care, which makes these people look for health services only when they are sick.

In the subcategory managers’ commitment, we found that the study participants had a negative perception of the managers’ commitment to the health of their communities, as most participants assessed it as terrible. This perception can be confirmed through the following statements:

“Bad. They treat us as invisible, they don’t look at us with good eyes, they don’t see our priorities and they don’t respect our rights as quilombolas”.

(Ci2)

“Terrible. The governor does not know the reality of the communities and the mayor is the one who lives in the city and knows our reality and does not care for our needs, even more with our health. We hardly go to the health center because it is far and there is no way to go”.

(Ca2)

“The manager does not make much effort for the community, we here report directly, talk, speak, and they do not do the monitoring that is necessary even from the municipality. That’s how they say: The policy won, it’s over”.

(G2)

## 4. Discussion

### 4.1. Characterization of Study Participants

After the results, the predominance of women in the interviewees is related to gender and the role of caregiver in the home and family. Due to their low level of education, they have fewer opportunities for work and social advancement. Another important finding was the preponderance of married people among those surveyed, which is an important issue, as the women who participated in the study have a source of support that they can have in difficult times and share daily tasks [40].

Regarding the low education level, scarcity of resources, and poor access to health services, studies show that the living conditions of quilombola communities show the situation of an individual, social, health, and programmatic vulnerability of this population [18,19,20,21]. Vulnerability is a word of Latin origin (vulnus), which means “wound”, commonly used to refer to the greater possibility that the individual has impaired well-being, quality of life and health, and, consequently, a higher risk of becoming ill [22,23].

In relation to income, Brazil corresponds to the largest economy in Latin America and the seventh economy in the world and, in the year 2019, presented per capita income of R$1439.00, although 7.4% of Brazilians are in extreme poverty. The country occupies the 10th position of a group of 143 countries in the inequality ranking. In terms of income concentration, Brazil represents the second country in the world with the greatest inequality in income distribution [41] (p. 6).

The Brazilian Institute of Geography and Statistics (IBGE) points out that black men and women (black and brown) are more limited in socioeconomic terms when compared to white men and women. Furthermore, blacks have an average per capita household income of R$934, compared to an average income of R$1846 for individuals who have color and/or are white [42].

As for infrastructure, studies reveal that black individuals remain living in areas and housing with poor infrastructure, in addition to greater exposure to epidemiological vectors, which are transmitters of disease among humans, or from animals to humans.

It is noteworthy that 12.5% of blacks live in areas without garbage collection and only 6% of the white population; without water supply by general network, blacks represent 17.9%, and whites 11.5%; without sanitary exhaustion by collecting or pluvial network, blacks represent 42.8% of the population against 26.5% of white

[42,43] (p. 5).

Thus, it is pointed out that the poor housing conditions, lack of basic sanitation, treated and quality water for human consumption, as well as the inadequate disposal of waste, poor access to health services show the vulnerability situation of this group, contributing to the high incidence and prevalence of chronic pathologies, such as: systemic arterial hypertension (SAH) and parasitic infections [26,44].

### 4.2. Knowledge about the Concept of Health

The study participants showed positive perceptions about the concept of health, as they referred to concepts that are close to those disseminated by WHO. Furthermore, they associated health not only with the physical dimension but with a set of factors (social, economic, cultural, environmental, access to health services, among others) that must be interconnected and, necessary for greater well-being, quality of life, and better health conditions for the individual and the community, as they are determinants for individual health conditions [26].

Unlike the findings of this study, surveys conducted with quilombolas from communities in the north of Minas Gerais and Vitória da Conquista showed a negative self-perception of health in the population they studied, associated with inequities in class, gender, race/ethnicity, access health services and the concept of health as the absence of disease [21,45].

### 4.3. Most Frequent Health Problems

We found that systemic arterial hypertension (SAH) is the disease that most frequently affects quilombolas in the northern Tocantins. This fact may be related to genetic issues, lifestyle, low education levels and, consequently, less practice of healthy habits, which can be aggravated by less access to health and care services at different levels of care, and also by the non-inclusion of this population in health promotion and disease prevention actions. The literature points out that SAH is one of the most incident and prevalent diseases among black men [46], responsible for a high number of deaths among men and women of this race [47].

A study on quality of life and metabolic syndrome conducted with 147 adult quilombolas from the central region of the State of Tocantins found that 48% of them had SAH [48]. In this same thought, a study on arterial hypertension and related factors in quilombola communities in Vitória da Conquista-BA also found high levels of blood pressure [49].

In addition to SAH and DM, sickle cell anemia was another pathology reported by quilombolas in the community of Cocalinho and its surroundings. Sickle cell anemia is a common pathology in quilombolas, which is related to genetic inheritance, and which presents specific clinical manifestations, with crises that cause pain, discomfort, and that require immediate medical assistance, as it has a high potential for complications, which compromises the health of the sick individual [50].

Sickle cell disease (SCD) comes from Africa and is found in 25% to 40% of African countries associated with changes in the hemoglobin gene due to stressful events, decreasing the blood oxygen tension [51]. Therefore, the erythrocyte assumes the shape of a sickle, a process called “sickling”, which obstructs the microcirculation vessels and the blood supply may be deficient or non-existent and, consequently, cause tissue death. In such a way, the research emphasizes that sickle cell anemia and arterial hypertension are problems that commonly affect the remaining quilombos [5,6,8].

### 4.4. Access to Health Services and Health Care

Regarding health care, we identified that the community of Cocalinho and surroundings has greater access to health and care services as revealed by one of the statements: “We have a care in the community, as the doctor is every Monday here, the dentist also comes and the nurse comes once or twice a week” (Co11). The other communities have low coverage of FHt. Similar results were found in a study carried out with quilombolas in the southwest of Bahia. Due to the low coverage of the FHS, the researchers emphasized the need for greater performance of these teams, to meet the health demands of the population they studied [52].

The literature states that, in the North and Northeast regions, the population has restricted access to health services, especially in primary health care due to the smaller number of medical professionals in these regions when compared with the assistance coverage of FHt in the Midwest regions, South and Southeast [53]. For this reason, the performance of these teams in the territory was essential since they enable greater access by quilombolas to health services, reducing the health inequities experienced by them [54].

The care offered to priority groups in the communities studied differs are different from the proposal by the Ministry of Health’s programs since this is centered on the hegemonic model with a curative focus and little insertion of the studied population in actions of health promotion and disease prevention. These findings reveal that comprehensiveness, health, and racial equity, as well as social control and universality proposed by SUS, are not being guaranteed.

We also found that the FHS is the service most used by the population studied, probably because it is the only service available in most of the cities studied, contributing to one of the essential attributes of PHC, the access. We also noticed that the assistance offered to quilombolas in the north of Tocantins is fragmented, centered on the biomedical model, which is to treat only the disease compromising the quality of care, the bond between the patient, the professional and the health service, the articulation of intersectoral actions, preventing the integrality of assistance and the guarantee of continuous care and the essential attributes of PHC: longitudinally, coordination, and integrality [55] Similar findings were found in the quilombola community of Buriti do Meio, Minas Gerais [54]. The FHt is responsible for the sanitary, nutritional conditions and, mainly, for the health of the population under their responsibility in the territory covered by the FHS [56].

Corroborating the findings of the study, research on the access and use of health services by quilombola women in promoting reproductive health found that the assistance provided to women during the pregnancy-puerperal cycle is weakened. This is because even when performing prenatal care, women experienced some difficulties in obtaining follow-up, such as distance, the health unit is far from the community; little knowledge about the importance of assistance during the pregnancy-puerperal cycle, and an unfavorable economic situation [20].

The reports also showed that health promotion actions were not carried out in five of the studied communities. This shows that the integrality of the assistance proposed by the Unified Health System is not being carried out since these actions are PHC priorities and essential pillars for the implementation of the expanded health concept.

Other studies also showed that the barriers (distance, social exclusion, lack of transportation, limited financial conditions, racism, lack of doctors, medication, and the obstacles for scheduling and conducting exams) faced by quilombolas in the North of Tocantins to have access to health services are also faced by quilombolas from different regions of the country. For example, in Bahia, Vitória da Conquista, Goiás, Amazonas, revealing not performing health as a right for that ethnic–racial group [44,52,57,58]. Several remaining quilombo communities are located in rural areas, far from the urban area. For this reason, location is a factor that negatively interferes with this population’s access to health services and, when they seek such services, do not receive quality care [19,52].

Other important points found in the study and deserving special attention are related to the negative perception of the researched population with the assistance received and the health services, with the managers’ commitment in terms of health. Diverging from the results of this study, research on the use of health services by quilombolas in southwestern Bahia, located in the northeastern region of Brazil showed that most participants evaluated the care received as good, although they underutilized health services, and associated the difficulties in accessing health services to social determinants [44].

Regarding the managers’ commitment, we emphasized that after the institution of SUS, responsibility for health started to be decentralized and managers from different federal, state, and municipal spheres assumed the responsibility of administering and offering health care services to resolutive and quality health to provide better health and living conditions for the population [58].

Furthermore, there is currently a topic widely discussed and with worldwide repercussions that put everyone’s health at risk, the Covid-19 pandemic, whose first cases were reported in Wuhan, China, in December 2019, as idiopathic pneumonia, which affected several Chinese, but which showed, even more, the inequities faced by the remaining quilombos. In Brazil, the first case of Covid-19 was diagnosed in February 2020, and the World Health Organization declared a state of public health calamity at the end of February. Covid-19 has already affected several quilombolas, from different regions of the country, such as those located in the states of Amapá, Bahia, Goiás, Pernambuco, and Rio de Janeiro, Rio Grande do Sul, and other regions of the country. Many quilombolas were infected and recovered, but some died due to lack of health information and also because they did not receive comprehensive health care, equally and with quality in their territories [25]. This information reinforces what many studies emphasize the lack of assistance and access to health services [45,46,47,48,52,57].

As a result, in September 2020, the National Coordination of Articulation of Black Rural Quilombola Communities (*CONAQ*) with the support of five (5) political parties, filed with the Supreme Court a request for the federal government to prepare a coping plan for Covid-19 in quilombola communities since the incidence of infected people and deaths were increasing considerably with each month that the pandemic extends. The plan involves the distribution of basic food baskets, personal protective equipment, personal hygiene materials, as well as providing clean water, greater access to hospital beds, and also reinforcing the need for FHt to be more active in the territories of these communities [59].

Reinforcing these arguments, Camara Jones, an African-American epidemiologist [60] (p. 3) stated that: “Blacks are becoming more infected by Covid-19 because they are more exposed and, once infected, they are dying more because their bodies—our bodies—carry the weight of the chronic lack of investment and the active neglect of the community”.

In this context, it should be noted that in Brazil the number of deaths per COVID-19 among black and brown individuals is much higher than the number of inhabitants per region, since 54.8% of the Brazilian population is black 39. Thus, when analyzing the deaths by VOCID19 it was found that 61% of these occurred among individuals of the black race. In the northern region of Brazil, the percentage of deaths by COVID-19 among blacks and mediums brown was 86%, where the population representation in this region and 76%. In the Northeast, the death rate was 82% for blacks, which constitute 70% of the total population of this region [14].

### 4.5. Study Limitations

The fact that the study was carried out in only six of the 44 quilombola communities in the state of Tocantins, and in a single period, limits the information related to health care offered to traditional communities in that state. However, the sample size used in the study was representative, mainly because it is qualitative research and because it exposes the subjectivity of the study individuals in health care, which is essential for individual and collective well-being and quality of life. We also observed the particularities of an ethnic-racial group, the most common health problems, and their demands, which makes this study a pioneer.

We also highlight the geographical location of the communities as limitations since most are located in the rural area, with the lack of transportation and the poor conditions of infrastructure (road, sanitation), a problem frequently experienced by the remainders and researchers since most of them lack asphalt pavement.

## 5. Conclusions

When assessing the health care offered to quilombola communities in northern Tocantins, we noticed that this population faces social and health inequalities, and greater exposure to social determinants of health, which consequently make them more susceptible to illness.

We also found that the scarcity of financial resources, the location of the communities, the lack of doctors, and difficulties in scheduling the exams, constitute barriers to the access of the studied population to health services. Moreover, the negative perception of the assistance received, and the commitment of the managers can negatively reflect on health indicators, and, above all, affect the quality of life and well-being of quilombolas in communities in the northern region of Tocantins.

Therefore, it is essential to prioritize the health of quilombolas and to expand the coverage of Family Health teams to assist in the territory of these communities to meet the health needs of this population and, to insert them in actions of promotion, prevention, and recovery of the sick individual, and, therefore, contemplating the principles of equity and integrality of SUS and attributes of primary health care. Managers must seek to reduce the existing inequalities, from the implementation of more efficient and resolving public policies that meet the needs of this population, and that provide better living and health conditions for quilombolas in Tocantins.

Finally, the results found in the study may foster new research to explore the theme addressed, knowing the reality of each quilombola community in the state of Tocantins and including other aspects that were not covered in this study.

## Figures and Tables

**Table 1:** Interview script to assess access to health services and assistance offered to remain quilombo communities in the northern region of the state of Tocantins, Brazil.

**Broad-Ranging Questions**
1—What is health for you?
2—How do you assess the health care offered to the population in your communities?
**Survey Questions**
1—Do you receive assistance in the territory?
2—What are the health problems that most affect quilombolas in this community?
3—Is there a family health team that assists within the community?
4—What are the difficulties faced to access health services?
5—In the most serious cases of illness, how are you treated?
6—Does the community health worker make a monthly visit to each home in the community? Are you satisfied with the work done by the community health worker? Justify yourself.
7—What do you suggest to improve the health care received by the community?
8—How do you assess the managers’ commitment to health care with this community?

**Table 2:** Categories and subcategories elaborated from the speeches of the participants. Northern Tocantins Region /Brazil, October 2017 to July 2018.

Category 01	Subcategory 01
Characterization of study participants	Gender; Age group; Education; Wage Income; Occupational Situation; Community infrastructure
**Category 02**	**Subcategory 02**
Most frequent health problems	Systemic Arterial Hypertension; Diabetes Mellitus; Worm infection; Sickle cell anemia.
**Category 03**	**Subcategory 03**
Knowledge about the concept of health	Positive health concept; Health as an essential condition for life.
**Category 04**	**Subcategory 04**
Access to health services and health care	Lack of assistance in the territory; Access barriers to health services; Fragmented assistance; Negative perception of health care; Lack of commitment by managers; Negative perception of managers’ commitment.

## Data Availability

Data available on request due to restrictions, e.g., privacy or ethical. The data presented in this study are available on request from the corresponding author.
